# The role of survivin in angiogenesis during zebrafish embryonic development

**DOI:** 10.1186/1471-213X-7-50

**Published:** 2007-05-18

**Authors:** Alvin CH Ma, Rachel Lin, Po-Kwok Chan, Joseph CK Leung, Loretta YY Chan, Anming Meng, Catherine M Verfaillie, Raymond Liang, Anskar YH Leung

**Affiliations:** 1 Department of Medicine, University of Hong Kong, Hong Kong; 2 Department of Biology and Chemistry, City University of Hong Kong, Hong Kong; 3 Department of Biological Sciences and Biotechnology, Tsinghua University Beijing, China; 4 Stem Cell Institute, University of Minnesota, Minnesota, USA

## Abstract

**Background:**

Survivin is the smallest member of the inhibitor of apoptosis (IAP) gene family. Recently, the zebrafish *survivin-1 *gene has been cloned, showing remarkable sequence identity and similarity over the BIR domain compared with human and mouse *survivin *gene. Here we investigated the role of survivin in angiogenesis during zebrafish development. Morpholinos (MOs) targeting the 5' untranslated region (UTR) (Sur_UTR_) and sequences flanking the initiation codon (Sur_ATG_) of zebrafish *survivin-1 *gene were injected into embryos at 1–4 cell stage. Vasculature was examined by microangiography and GFP expression in *Tg(fli1:EGFP)*^*y*1 ^embryos. Results: In embryos co-injected with Sur_UTR _and Sur_ATG_-MOs, vasculogenesis was intact but angiogenesis was markedly perturbed, especially in the inter-segmental vessels (ISV) and dorsal longitudinal anastomotic vessels (DLAV) of the trunk, the inner optic circle and optic veins of developing eyes and the sub-intestinal vessels. Apoptosis was increased, as shown by TUNEL staining and increase in caspase-3 activity. Efficacy of Sur_UTR _and Sur_ATG_-MOs was demonstrated by translation inhibition of co-injected 5'UTR survivin:GFP plasmids. The phenotypes could be recapitulated by splice-site MO targeting the exon2-intron junction of *survivin *gene and rescued by *survivin *mRNA. Injection of human vascular endothelial growth factor (VEGF) protein induced ectopic angiogenesis and increased survivin expression, whereas treatment with a VEGF receptor inhibitor markedly reduced angiogenesis and suppressed survivin expression. Conclusion: Survivin is involved in angiogenesis during zebrafish development and may be under VEGF regulation.

## Background

Survivin is the smallest member of the inhibitor of apoptosis (IAP) gene family containing a single Baculovirus IAP Repeat (BIR) domain and an extended -COOH terminal α-helical coiled coil [1]. Survivin is not expressed in most normal adult tissues but is highly expressed in solid and hematological malignancies, where it has been linked to increased angiogenesis and tumorigenesis [2,3]. During human and murine embryonic development, survivin is ubiquitously expressed [4]. However, homozygous knock-out of *survivin *in mouse ES cells results in disrupted microtubule formation and polyploidy as well as early embryonic fatality, precluding characterization of its functions during murine development [5]. As a result, the role of survivin during embryonic development remains unclear.

Recently, the zebrafish *survivin-1 *gene (abbreviated *survivin*) has been cloned, showing remarkable sequence identity and similarity over the BIR domain compared with human and mouse *survivin *gene [6]. Microarray analysis showed that *survivin *is significantly up-regulated in a zebrafish *chordin *morphant in which the intermediate cell mass (ICM, where vascular and primitive hematopoietic tissues arise) was expanded [7]. Here, we investigated if survivin plays a role in vascular formation during zebrafish embryonic development.

## Results

### Expression of survivin in zebrafish embryos

**Figure 1:**
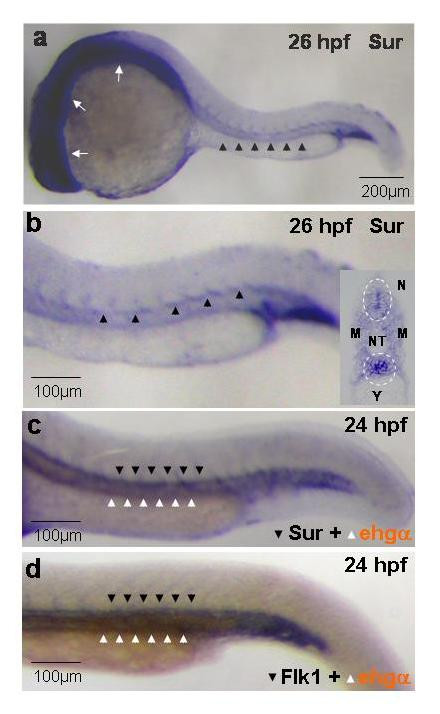
Whole-mount in-situ hybridization showing the expression of *survivin *in zebrafish embryos. (a, b): *Survivin *is expressed diffusely in the developing central nervous system (white arrows) and the axial vasculature (arrowheads) at 26 hpf. Similar expression patterns were seen at 56 hpf (not shown). (b, insert): Coronal section of stained embryos at 26 hpf showing preferential expression of *survivin *at the vicinity of the dorsal aorta and the developing neural tube (circled). (c, d): Double *in-situ *hybridization showing remarkably similar expression pattern of *survivin *(c) and *flk1 *(d) (blue, dark arrowheads) in relation to that of *embryonic hemoglobin-α *(brown, white arrowheads). Pictures are representative of at least three separate experiments. NT: Neural Tube; M: Myotome; N: Notochord; Y: Yolk sac extension.

Whole-mount *in-situ *hybridization was performed to examine *survivin *mRNA expression in zebrafish embryos at 26 hpf. *Survivin *was detected diffusely throughout the developing brain and neural tube. It was also expressed at the vicinity of the axial vasculature from which the inter-segmental vessels arise (Figure 1a–b). This was further confirmed in histological sectioning in which the areas corresponding to the developing axial vasculature and neural tube showed increased expression relative to the adjacent tissues (Figure 1b, insert). Furthermore, double *in-situ *hybridization showed that *survivin *was expressed in the developing axial vasculature dorsal to the intermediate cell mass (ICM), where gene encoding for embryonic hemoglobin α was expressed. The pattern was remarkably similar to that of *flk1*, a VEGF receptor tyrosine kinase (Figure 1c–d).

### Survivin morphants

**Figure 2:**
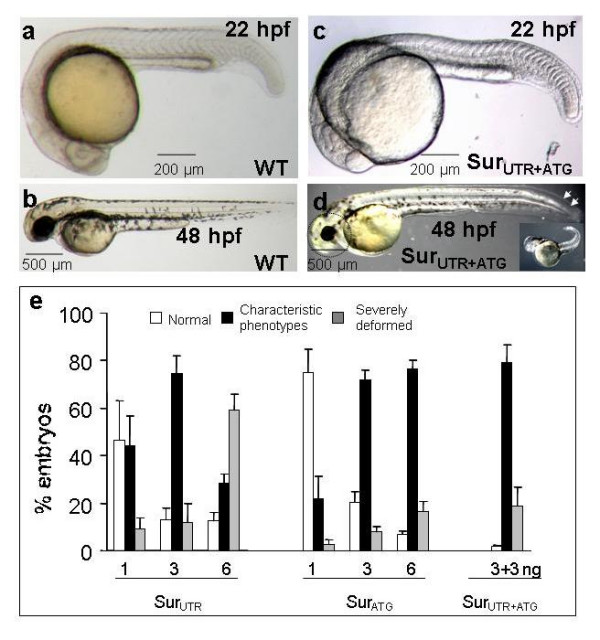
Effects of *survivin *knock-down on embryonic development. (a, b): Wild-type (WT) embryos injected with random sequence morpholino at 22 hpf (a) and 48 hpf (b). (c, d): Embryos injected with a combination of Sur_ATG _(3 ng) and Sur_UTR _morpholinos (3 ng) (Sur_ATG+UTR_) at 1–4 cell stage. Noted that while there was no significant morphological changes at 22 hpf, most of the embryos injected with Sur_ATG+UTR _morpholinos at 48 hpf showed a "characteristic phenotype" with reduced head and eye size (circled) and a mildly curved tail (arrowheads). Similar phenotypes were also seen in embryos injected with either Sur_ATG _or Sur_UTR _morpholinos at various doses but not in WT embryos injected with random sequence. Insert (d) showed a severe phenotype at 48 hpf characterized by severely deformed embryos which did not survive beyond 48 hpf. These embryos were not included in the analysis. Each picture is representative of at least three experiments. (e): The dose-dependence of either Sur_ATG_, Sur_UTR _or Sur_ATG+UTR _morpholinos. Optimal response was observed when embryos were co-injected with 3 ng of each MO (Sur_UTR+ATG_). Results were expressed as mean ± S.E.M. In each experiment, MOs at different doses were injected into the same batch of embryos and were scored at the same time. More than 40 embryos have been injected at each dosage.

The role of survivin during embryonic development was investigated by knocking-down its function using MOs. The phenotypic penetrance of *survivin *MOs was dose- and time-dependent. At 22 hpf, when injected with either 3 ng Sur_UTR _or 3 ng Sur_ATG_-MOs (referred as Sur_UTR_^mo ^and Sur_ATG_^mo ^embryos), most embryos had a relatively normal morphology (Figure 2a,c). However, at 48 hpf, 74.8 ± 7.3% and 72.0 ± 4.0% of embryos manifested "characteristic phenotypes" with reduced eye and head sizes and a mildly curved tail (Figure 2b,d). There was no overt tissue necrosis in these embryos. At 6 ng of either MOs, increasing numbers of embryos became severely deformed and died shortly after 48 hpf (Figure 2d, insert). Co-injecting Sur_ATG _+ Sur_UTR_-MOs (3 ng each) resulted in specific phenotypes in 79.4 ± 7.2% embryos without increase in toxicity or mortality as compared with 3 ng of either MO alone (Figure 2e). The combination regimen remained significantly less toxic than that of Sur_UTR_-MO at 6 ng. In all subsequent experiments, Sur_ATG _and Sur_UTR_-MOs were co-injected at 3 ng each (referred as Sur_UTR+ATG_^mo ^embryos). Only embryos with characteristic phenotypes were investigated while those which were severely deformed were excluded from analysis.

### Effects of survivin knock-down on angiogenesis

**Figure 3:**
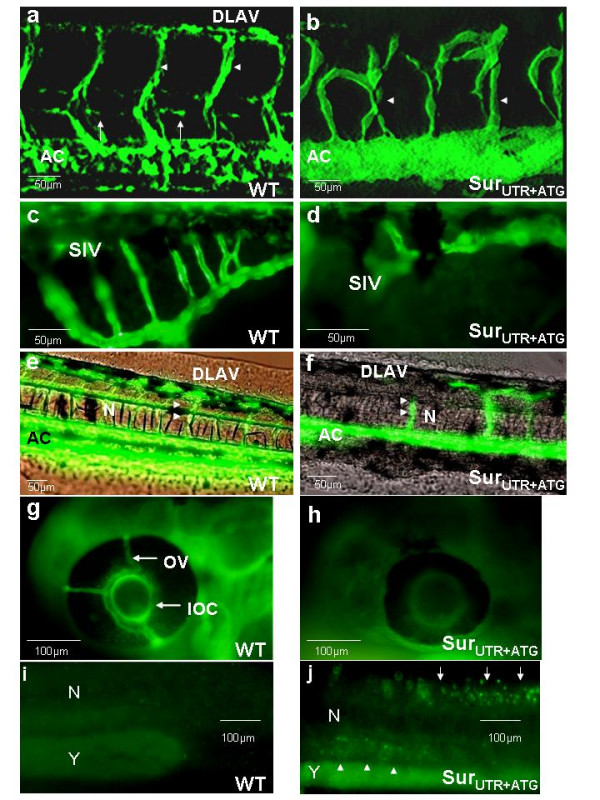
Effects of *survivin *knock-down on angiogenesis and circulation. (a, b): Confocal microscopy of *Tg(fli1:EGFP)*^*y*1 ^embryos either uninjected (a) or injected with Sur_UTR+ATG _morpholinos (b). Noted the aberrant sprouting of the inter-segmental vessels (ISV) (arrowheads), the absence of vertebral arteries (arrows) and the failure to form the dorsal anastomotic vessels (DLAV) in the Sur_UTR+ATG_^MO ^embryos. AC: Axial circulation. Noted that the dorsal aorta and posterior cardinal vein in the axial circulation could not be distinguished based on the resolution provided. (c, d): Fluorescent images in *Tg(fli1:EGFP)*^*y*1 ^embryos showing failure to develop the sub-intestinal vessels (SIV) in Sur_UTR+ATG_^MO ^embryos. (e-h): Microangiographic pictures in uninjected (e, g) and Sur_UTR+ATG_^MO ^embryos (f, h) showing defective vasculatures in ISV, DLAV, optical veins (OV) and inner optic circle (IOC). N: Notochord; AC: Axial circulation. (i, j): Whole-mount TUNEL assay in embryos injected with random sequence MO (i) and Sur_UTR+ATG_-MO (j) showing positive staining in the area of developing neural tube and brain (white arrows) as well as at the vicinity of the axial circulation (white arrowheads) in the Sur_UTR+ATG_^MO ^embryos. N: Notochord; Y: Yolk sac extension. Embryos were examined at 48 hpf except (c) & (d) which were examined at 96 hpf. More than 20 embryos have been examined in each experiment.

We have previously shown that *survivin *is significantly up-regulated in a zebrafish chordin morphant in which the ICM was expanded [7]. Therefore, we first examined the effects of *survivin *knock-down on vascular formation in *Tg(fli1:EGFP)*^*y*1 ^embryos. In uninjected embryos, the axial circulation (AC), inter-segmental vessels (ISV), dorsal longitudinal anastomotic vessels (DLAV), vertebral and sub-intestinal vessels (SIV) were readily observable (Figure 3a,c). In Sur_UTR+ATG_^mo ^embryos, the dorsal aorta and posterior cardinal vein were patent, indicative of intact vasculogenesis (see additional file 1: Wild-type embryos and file 2: Survivin morphants). However, the development of vertebral and ISV was perturbed with defective or total absence of sprouting as well as failure to form the DLAV and SIV (Figure 3b,d). These defects were seen in all 54 Sur_UTR+ATG_^mo ^embryos observed (n = 3 separate experiments) with the characteristic phenotypes. The results were confirmed using microangiography in which defective ISV sprouting and failure to form the DLAV, as well as defective inner optic circle (IOC) and optic veins (OV) of the developing eyes were seen in the Sur_UTR+ATG_^mo ^embryos (Figure 3e–h). Similar patterns of angiogenesis defects were observed when either Sur_UTR _or Sur_ATG _morpholinos were injected (data not shown).

### Effects of survivin MOs on apoptosis as shown by TUNEL and caspase-3 activity

As a member of the IAP family, survivin has been shown to inhibit apoptosis by regulating caspase activity [1,2]. Therefore; we investigated if there was increased apoptosis in the Sur_UTR+ATG_^mo ^embryos as measured by TUNEL assay. At both 24 and 48 hpf, increased TUNEL staining was detected in the developing neural tube and the brain (not shown), with significant, albeit weaker, staining at the vicinity of the axial vasculature (Figure 3i,j). The increased apoptosis was further confirmed by specific caspase-3 activity which was significantly increased in 48 hpf Sur_UTR+ATG_^mo ^embryos (299.1 ± 8.3 arbitrary units) compared with control embryos injected with a random sequence MO at 6 ng (103.0 ± 2.3 arbitrary units, n = 3 experiments using 240 embryos, p < 0.05).

### Specificity of survivin knock-down

**Figure 4:**
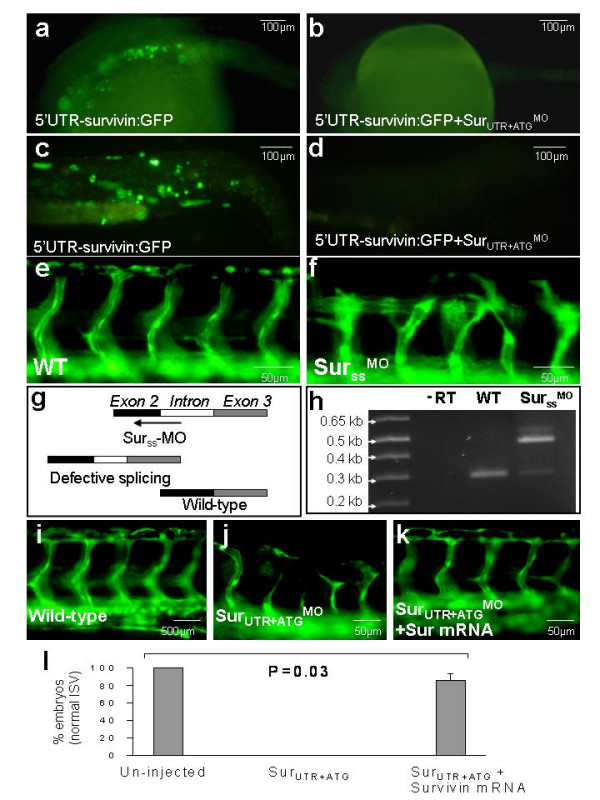
Effect of *survivin *knock-down was gene-specific. (a-d): Injection of 5'UTR-survivin:GFP plasmids gave rise to green fluorescence in a mosaic pattern in 79.7 ± 9.4% (a, c) which was totally abolished by co-injection with Sur_UTR+ATG_-^MO ^(b, d). (e): Uninjected *Tg(fli1:EGFP)*^*y*1 ^embryos at 48 hpf. (f): Defective sprouting of inter-segmental vessels, similar to those seen in Sur_UTR+ATG_^MO ^embryos, could be recapitulated by injecting embryos with *survivin *morpholino targeting the splice-site junction (g). (h): Molecular targeting was confirmed using RT-PCR showing *survivin *gene in injected embryos contained a larger transcript compared with uninjected ones. (i-k): Defective angiogenesis in Sur_UTR+ATG_^MO ^embryos could be rescued by co-injecting with *survivin *mRNA. (i): Uninjected embryos. (j): Sur_UTR+ATG_^MO ^embryos. (k): Sur_UTR+ATG_^MO ^embryos co-injected with *survivin *mRNA. (i): Histogram showing average number of embryos with normal inter-segmental vessels (ISV) in three separate experiments. All embryos were oriented anterior (left) to posterior (right).

To further demonstrate the efficacy of Sur_UTR _and Sur_ATG _MO binding to *survivin *mRNA, embryos were co-injected with a 5'UTR survivin:GFP plasmid (50 pg) and Sur_UTR+ATG_-MOs (3 ng each). Injecting the plasmid alone lead to GFP expression in 79.7 ± 9.4% embryos (Figure 4a,c). Co-injection of the plasmid with Sur_UTR+ATG_-MOs completely abolished protein translation and hence GFP expression in all embryos tested (Figure 4b,d). A splice site MO (Sur_SS_-MO (12 ng)) not only induced similar morphological changes as in Sur_UTR+ATG_^mo ^embryos (smaller head and eye size and mildly curved tail) but also induced defective angiogenesis as shown in *Tg(fli1:EGFP)*^*y*1 ^embryos (61.7%, n = 3 experiments using 159 embryos) (Figure 4e–f). Angiogenesis defects were seen in ISV as well as OV/IOC of the developing eyes (not shown). A relatively high dose of MO (12 ng) was used as lower doses produced less phenotypic penetrance and at 12 ng, there was no excessive mortality. In the Sur_SS_^MO ^embryos, RT-PCR confirmed defective splicing of part of the intron, as shown by a larger PCR transcript which was verified by bi-directional DNA sequencing (Figure 4g,h). Whether defective splicing could be induced by lower doses of this MO has not been examined. Finally, defective sprouting or failure to form the DLAV occurred in all Sur_UTR+ATG_^mo ^embryos and co-injecting *survivin *mRNA (30 pg) with Sur_UTR+ATG_-MOs rescued the vascular defect in 47 out of 58 embryos in three separate experiments (81%) (Figure 4i–l).

### Effects of VEGF on survivin expression

**Figure 5:**
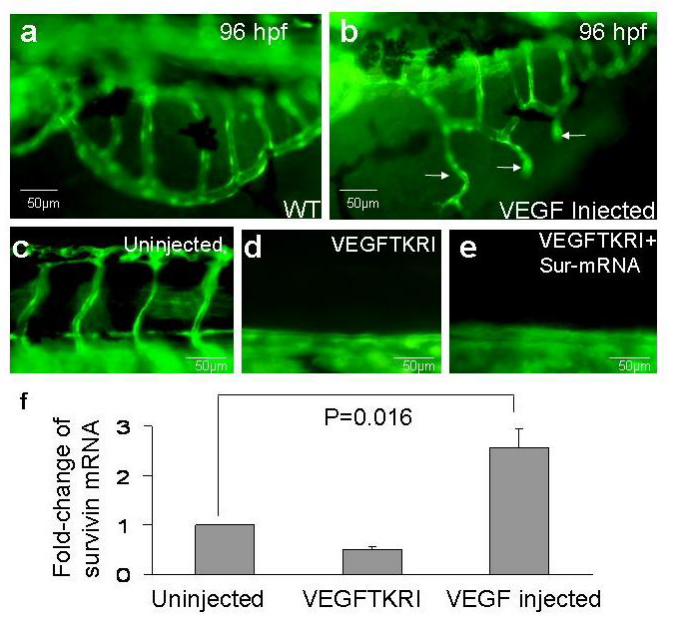
Regulation of survivin expression by vascular endothelial growth factor (VEGF) at 96 hpf. (a): Sub-intestinal vessels in uninjected *Tg(fli1:EGFP)*^*y*1 ^embryos. (b): Injection of human VEGF (2 ng) gave rise to ectopic angiogenesis (arrows). There was no observable ectopic angiogenesis in the ISV (c): Axial and inter-segmental vessels in untreated *Tg(fli1:EGFP)*^*y*1 ^embryos. (d): *Tg(fli1:EGFP)*^*y*1 ^embryos treated with VEGF tyrosine kinase receptor inhibitor (VEGFTKRI, 25 μmol/L) showing defective sprouting of inter-segmental vessels. (e): Injection of *survivin *mRNA did not reverse the defects seen in VEGFTKRI treated embryos. (f): Histogram showing the average *survivin *mRNA expression (expressed in fold-change) in untreated and VEGFTKRI treated embryos as well as in embryos injected with human VEGF. Results expressed in mean ± S.E.M. (n = 3 experiments using at least 20 embryos per experiments). When the three groups of data were compared using Kruskal-Wallis Test, p-value = 0.016. When the data of uninjected *vs *VEGFTKRI treated embryos were compared using Mann-Whitney U Test, p-value = 0.037.

VEGF plays an important role in angiogenesis during zebrafish embryonic development [8]. *In-vitro *studies have shown that survivin mediates the proliferative and anti-apoptotic effects of VEGF in endothelial cells [9]. Therefore; we investigated if *survivin *expression during embryogenesis is regulated by VEGF. Exogenous human VEGF protein (2 ng) was injected into zebrafish embryos at one-cell stage [10]. Angiogenesis was examined in the sub-intestinal vessels at 96 hpf, where the vasculature was well-developed and any ectopic structures could be readily detectable. In 78 out of 110 embryos (70%) (from three separate experiments), VEGF induces ectopic angiogenesis which was associated with a significant up-regulation of *survivin *mRNA expression (Figure 5a–b,f). We also incubated embryos with a VEGF receptor inhibitor (VEGFTKR) at one-cell stage. VEGFTKR (25 μmol/L) induced defective angiogenesis in all treated embryos at 48 hpf (Figure 5c–d) and could not be rescued by *survivin *mRNA injection (30 pg) (Figure 5e). *Survivin *mRNA expression was significantly down-regulated in these embryos (Figure 5f).

## Discussion

In this study, we demonstrated that knock-down of *survivin *with MOs gives rise to embryos with reduced eye and head sizes and a mildly curved tail. Similar phenotypic changes have been described in a survivin mutant generated in a retrovirus insertional mutagenesis screen [11]. The *survivin *morphants had defective angiogenesis but vasculogenesis, i.e. formation of axial vasculature, was not affected at the doses of MOs tested. Development delay in these morphants was not observed, as shown by the normal onset and pattern of pigmentation and heart beat (data not shown). Our results corroborate with *in-vitro *studies showing that survivin is important for the maintenance of proliferation and survival on endothelial cells [9,12]. In addition, our data provided new information on the role of survivin during embryonic development.

*In-vitro *and tumorigenesis studies have shown that survivin mediates the angiogenic effects of VEGF [9,12,13]. In zebrafish embryos, VEGF signaling is important for angiogenesis. In particular, mutants defective in a zebrafish orthologue of flk1 (a VEGF-receptor tyrosine kinase), the *schwentine *[14], and in phospholipase Cγ (a tyrosine kinase mediating effects of VEGFR), the *y10 *[15], exhibit specific defects in angiogenesis. MO targeting of VEGF results in defective circulation in the head, axial and inter-segmental vasculature in a dose-dependent fashion [8]. In this study, VEGF induces ectopic angiogenesis and up-regulates *survivin *mRNA expression, suggesting that survivin may mediate the angiogenic effect of VEGF. The link between VEGF and survivin during zebrafish angiogenesis has not been examined but may involve PKB/Akt signaling as reported in human endothelial cell lines [16]. Intriguingly, co-injecting embryos with survivin mRNA could only rescue the vascular defects seen in Sur_UTR+ATG_^MO ^embryos but not in embryos treated with a VEGF receptor inhibitor. Therefore, additional downstream mediators may be involved in the angiogenic effects of VEGF [17]. Reversely, whether VEGF can rescue the angiogenesis defects in Sur_UTR+ATG_^MO ^embryos has not been examined. Perturbation of VEGF signaling may also result in changes in blood vessel synthesis and the observed changes in survivin mRNA may reflect changes in endothelial cell number rather than a direct mechanistic link to VEGF signaling. This issue would have to be evaluated in future study.

Both human and murine studies have demonstrated that survivin is involved in haematopoietic stem and progenitor cell proliferation [18,19]. However, in the present study, early specification of hematopoietic progenitors in the Sur_UTR+ATG_^MO ^embryos was not affected, as shown by the normal expression of genes encoding for hematopoietic transcription factors and embryonic hemoglobins, as well as the normal distribution of gata1^+ ^population in *Tg(gata1:GFP) *embryos at 18 hpf, before the onset of functional circulation (data not shown).

That the targeting of the *survivin *MOs was specific was shown using several control studies. First, the phenotypic changes seen in Sur_UTR+ATG_^MO ^embryos were similar to those observed in *survivin *mutants generated by retrovirus insertional mutagenesis screening [11]. Indeed, it would be valuable to examine this mutant for similar defects in angiogenesis. Second, co-injecting Sur_UTR+ATG_-MO with a 5'UTR-survivin:GFP plasmid inhibited translation and hence green fluorescence induced by the latter in all embryos, proving efficacious binding of Sur_UTR+ATG_-MO to the 5'UTR-survivin mRNA. Third, the angiogenesis defects of ISV induced by Sur_UTR+ATG_-MO could be rescued by *survivin *mRNA. Whether the defects in SIV and OV/IOC were similarly reversed and whether injection of survivin mRNA alone would induce angiogenesis defects would have to be further examined. Finally a splice-site morpholino recapitulated the phenotypes seen with Sur_UTR+ATG_-MO. Therefore, the angiogenesis defects in Sur_UTR+ATG_^MO ^embryos represent a specific phenotype due to knock-down of *survivin *function in zebrafish embryos.

In human, murine [4] as well as Xenopus embryos [20], *survivin *is ubiquitously expressed. These observations, together with the fact that the developing head and eye of the Sur_UTR+ATG_^MO ^embryos were reduced in size have raised a concern whether the angiogenesis defects in these embryos might be caused by a general developmental defect and cell death, rather than a specific requirement for survivin function. However, in zebrafish embryos, *survivin *is more robustly expressed in the axial vasculature as well as the developing brain and neural tube. This observation has been confirmed by histological sectioning as well as double *in-situ *hybridization in which the pattern of *survivin *expression showed remarkable similarity to that of *flk1*, a marker of vascular endothelium. We cannot exclude a low level but diffuse expression of *survivin *in adjacent tissues but this should not negate the specific role of survivin during angiogenesis. First, we have confined our examination to the Sur_UTR+ATG_^MO ^embryos with characteristic phenotypes and none of them showed overt tissue necrosis at 48 hpf. The 20% embryos with severe deformity have been excluded from analysis. Second, we demonstrated that embryonic development is not overtly delayed in the Sur_UTR+ATG_^MO ^embryos as shown by the normal onset and pattern of pigmentation and heart beat (data not shown). Finally, in the present study, survivin expression appeared to be under VEGF regulation, providing a possible link between VEGF and embryonic angiogenesis. Our findings were also consistent with those by Pasquier et al. [20] in which over-expression of survivin in Xenopus embryos induces endothelial cell proliferation *in-vivo*.

Several observations in this study have remained unexplained. For instance, we did not observe a direct causal link between increased apoptosis and the angiogenesis defect in the Sur_UTR+ATG_^MO ^embryos. Apoptosis was detectable not only in the axial vasculature, but also in the developing brain and neural tube of the Sur_UTR+ATG_^MO ^embryos. Both *in-vivo *and *in-vitro *studies have demonstrated that in addition to its anti-apoptotic function, survivin plays an important role in the regulation of cellular proliferation and cytokinesis [1,2]. Recent study in Xenopus embryos also showed that *survivin *expression induces endothelial cell proliferation independent of apoptosis [20]. Therefore, the relative modest TUNEL staining in the axial vasculature did not preclude the role of survivin in angiogenesis. It is also possible that survivin plays a non-cell autonomous role in the angiogenesis process. Childs et al. (2002) [21] demonstrated in zebrafish embryos the migration of angioblasts from the aorta to the dorsal aspect of the neural tube and to the inter-phase between notochord and the somites, where they develop into DLAV and ISV. Therefore, vascular patterning may depend on signaling cues that direct the site of angiogenesis sprouts. Whether the occurrence of apoptosis in the developing neural tube might have perturbed these signals hence the formation of DLAV and ISV would have to be carefully examined. The proposition may also explain the lack of robust expression at the site of ISV and DLAV in wild-type embryos. Furthermore, although *survivin *is expressed robustly in the axial vasculature, concomitant expression was noted in the developing central nervous system. The expression of survivin within these structures needs to be defined in future study. Moreover, the developing eye and head structures in the survivin morphants are generally smaller. Whether this reflected changes secondary to defective angiogenesis or alternative functions of survivin during development have not been elucidated. Finally, survivin gene in zebrafish has undergone duplication during evolution [22] and the function of the duplicated gene would have to be further investigated. Notwithstanding these limitations, our data still supported the proposition that survivin is involved in the regulation of angiogenesis during zebrafish development.

*Survivin *is strongly expressed in both solid organ and hematological malignancies where it is associated with treatment failure and a poor prognosis [2,3]. Loss of function studies have also demonstrated that *survivin *expression is linked to angiogenesis and tumorigenesis in gastric and colonic cancers and has become a potential target for anti-cancer therapy [23,24]. Our observation that survivin regulates angiogenesis in zebrafish embryos highlights the relevance of using zebrafish embryos in the screening for survivin-based anti-cancer agents.

In summary, we demonstrate that survivin plays an important role in angiogenesis during embryonic development and may be one of the down-stream effectors of VEGF signaling. Early hematopoiesis was not affected but the role of survivin during late hematopoiesis remains to be determined.

## Methods

### Zebrafish and morpholinos

Wild-type zebrafish (*Danio rerio*) were obtained from local aquarium and were maintained and raised under standard conditions at 28°C. Transgenic *Tg(fli1:EGFP)*^*y*1 ^embryos were used to track endothelial cell populations. Anti-sense morpholinos (MO) (Gene-Tools, OR, USA) were designed to target the 5'untranslated region (UTR) or sequences flanking and including the initiation codon (ATG) of the zebrafish *survivin *gene. A splice-site (SS) MO was designed to target the exon2-intron junction of the *survivin *gene (Sur_SS_-MO). A random sequence MO was used as a control as described previously (Table 1). Procedures for micro-injection, whole mount in-situ hybridization, microangiography, TUNEL and caspase-3 activity assays have been described previously [7,25,26].

**Table 1:** Sequences of oligos used.

**Oligo**	**Sequence**
Morpholinos	
Sur_ATG_	TGC AAG ATC CAT TTT GTG GGA GGT T
Sur_UTR_	GTG GAA ATT AAA CAA AAG ACA ACC G
Sur_SS_	AGA CAC GGA CTC ACT CAG GGT CAT C
Random Sequence	CCT CTT ACC TCA GTT ACA ATT TAT A
	
Primers for the cloning of *survivin *mRNA in riboprobe synthesis	
ZF Sur_f_	CGG ATT TAT CTC GGT TGT CTT T
ZF Sur_r_	CAA CTT TCA CAA GTG ACA GAA CAC
	
Primers for the cloning of *survivin *UTR for 5'UTR survivin-GFP construct synthesis	
ZF SurUTR_f_	GCG GAT TTA TCT CGG TTG TCT
ZF SurUTR_r_	CTT CCT CCC CCA TCG CAG TCT GG
	
Primers for RT-PCR for *survivin *mRNA in splice-site morpholino study	
ZF Sur_f_	CAA CCT CCC ACA AAA TGG AT
ZF Sur_r_	GTC CAC AGT CTT CTT CAG CA
	
Primers for the cloning of *survivin *mRNA in rescue experiments	
ZF Sur_f_	AAT CAA CAA GCA AGCGAG AC
ZF Sur_r_	CAA TTT ATT AAG CCC GAA TGC
	
Primers for real-time quantitative RT-PCR for *survivin *mRNA	
ZF Sur_f_	CAC TCC AGA AAA CAT GGC TAA A
ZF Sur_r_	CCA TCC TTC CAG CTC TTT CA

### Double in-situ hybridization

Wild-type (WT) embryos at 24 hpf were fixed with 4% paraformaldehyde (PFA) and dehydrated. After stepwise re-hydration, the embryos were incubated in pre-hybridization buffer (50% formamide, 5 × SSC, 50 μg/ml heparin, 0.1% Tween20, 5 mg/ml rRNA in phosphate-buffered saline, PBS) at 65°C followed by overnight incubation with digoxigenin (DIG)- (either *flk-1 *or *survivin-1*) and fluorescein-labeled riboprobe (α-embryonic globin) at 65°C. The embryos were washed and incubated with alkaline phosphatase (AP) conjugated anti-DIG antibody (Roche Molecular Biochemicals, Mannheim, Germany) for overnight at 4°C. Blue color was developed using NBT/BCIP (Roche Molecular Biochemicals, Mannheim, Germany) as substrate and the reaction was stopped with 0.5 mM EDTA in PBT. AP was destroyed by washing the stained embryos with 0.1 M glycine-HCl, pH 2.2 in PBT for 10 min twice. Background staining was removed by washing the embryos in absolute ethanol with continuous monitoring. After re-hydration to PBT, embryos were incubated with AP conjugated anti-fluorescein antibody (Roche Molecular Biochemicals, Mannheim, Germany) for overnight at 4°C and red color was developed using INT/BCIP (Roche Molecular Biochemicals, Mannheim, Germany) as substrate.

### Synthesis of anti-sense mRNA riboprobe for survivin

The full length sequence of zebrafish *survivin *including the 3' UTR was amplified by PCR (Table 1) from cDNA of 24 hpf embryos and subcloned into pGem-T vector (pGEM-T Vector Systems, Promega, Madison, WI, USA). A 623 bp anti-sense *survivin *mRNA riboprobe was synthesized from linearized vector containing the insert. A digoxigenin labeled mRNA probe was synthesized by SP6 RNA polymerase according the manufacturer's protocols (Roche Applied Science, Indianapolis, IN, USA). The size and integrity of the synthesized riboprobe was confirmed by RNA formaldehyde gel electrophoresis. Histological assessment of stained embryos was performed on 5–7-μm paraffin sections.

### Construction of 5'UTR-survivin:GFP plasmids

The 5'UTR of *survivin*, including the target sequences of Sur_UTR_-MO and Sur_ATG_-MO, were amplified from 24 hpf wild-type embryo cDNA (Table 1). PCR products were gel purified and cloned in frame and immediately upstream of the GFP coding sequence into vector pcDNA3.1/CT-GFP-TOPO (Invitrogen, Carlsbad, CA, USA) and transformed into chemically competent *E. coli *TOP10 (Invitrogen, Carlsbad, CA, USA). Plasmids containing the 5'UTR-survivin:GFP fusion sequence were isolated and the sequence of the DNA inserts verified using the GFP reverse primer (5'-GGG TAA GCT TTC CGT ATG TAG C-3').

### Preparation of survivin mRNA for rescue experiments

The complete coding sequence of *survivin *was TA-cloned into pGEM-T vector (pGEM-T Vector Systems, Promega, Madison, WI, USA) and the orientation of the insert confirmed by PCR (Table 1). mRNA transcripts were synthesized from the T7 promoter of the *Sal *I digested pGEMT-Sur sequence using the mMessage mMachine Kit (Ambion, Austin, TX, USA).

### Treatment of embryos with VEGF receptor tyrosine kinase inhibitors

Embryos were treated with an inhibitor of vascular endothelial growth factor receptor tyrosine kinase (VEGFRTK inhibitors, Calbiochem, EMD Bioscience, CA, USA). The embryos were incubated in inhibitor solution at 25 μmol/L (stock solution in DMSO at 10 mmol/L) from one-cell stage onwards. They were dechorionated at 24 hpf with continuous exposure to inhibitors until 48 hpf. Control experiments were set up from the same clutches of embryos and were exposed to equal volume of DMSO for comparison.

### Vascular endothelial growth factor (VEGF) injection

Human VEGF protein (BD Bioscience, Bedford, MA, USA) was prepared in 1 mg/mL in water. Embryos at 1–4 cell stage were injected with VEGF (2 ng) into the yolk sac and its effect on angiogenesis was examined at 96 hpf.

### Real-time quantitative RT-PCR (Q-PCR)

cDNA from 48 and 96 hpf embryos were reverse transcribed from RNA and Q-PCR for *survivin *was performed using the SYBR green PCR master mix (Applied Biosystems, Foster City, CA, USA). Expression level was presented as fold-change calculated using the comparative C_T _method as described [27] with *β-actin *as the internal reference. Primer sequences for Q-PCR were shown in Table 1.

### Statistical analysis

Results were expressed as mean ± SEM unless otherwise stated. Comparisons between groups of data were evaluated by Mann-Whitney U and Kruskal-Wallis Test where appropriate. P-value of less than 0.05 was considered statistically significant.

## Authors' contributions

ACHM carried out the microinjection and molecular studies and wrote the manuscript. RL carried out the microinjection in some experiments. PKC carried out the confocal microscopy. JL and LC performed the histological sectioning of embryos. AM, CV and RL participated in the design of the study. AYHL conceived of the study, and participated in its design and coordination and wrote the manuscript. All authors read and approved the final manuscript.

## Supplementary Material

Additional File 1 Blood circulation in wild-type embryos. In wild-type embryos, normal axial circulation is observed as well as circulation in ISV and DLAV. Click here for file

Additional File 2 Blood circulation in survivin morphants. In survivin morphants, normal axial circulation is observed but circulation in ISV and DLAV was absent. Click here for file
